# Molecular and cell-biological mechanisms of antigen cross-presentation

**DOI:** 10.3389/fimmu.2013.00051

**Published:** 2013-02-28

**Authors:** Christian Kurts

**Affiliations:** Institute of Molecular Medicine and Experimental Immunology, Friedrich Wilhelms-Universität BonnBonn, Germany

Cross-priming serves to activate cytotoxic T lymphocytes for immune defense against viruses and tumors and plays an important role in vaccinations. Only certain dendritic cell (DC) subsets can cross-present. Several cell surface markers have been described that more or less specifically and sensitively characterize these subsets. The cell-biological mechanism(s) why a DC subset can cross-present are less clear. Theoretically, the task of cross-presentation can be divided into several mechanistic steps: (1) Antigen uptake by various endocytosis mechanisms, (2) Intracellular antigen routing into distinct organelles including the crossing of organelle membranes, (3) Antigen processing into peptides, (4) Peptide loading onto MHC molecules, and (5) Transport of these complexes to the cell surfaces for presentation to T cells. Each of these steps is dependent on numerous parameters, not only the DC subtype, but also the nature of the antigen or the presence of further signals that impact DC function or signify the presence of danger or infection.

This research topic contains 10 articles by leading experts in the field of antigen presentation that cover our current knowledge on the molecular mechanisms underlying cross-presentation (Chopin et al., 2012; Compeer et al., 2012; Harriff et al., 2012; Kreer et al., 2012; Kroczek and Henn, 2012; Murshid et al., 2012; Neefjes and Sadaka, 2012; Saveanu and van Endert, 2012; Thacker and Janssen, 2012; Wagner et al., 2012). The authors describe the influence of endocytosis receptors or heat shock proteins for antigen uptake, the intracellular logistics of antigen routing, membrane translocation mechanisms and proteases, the transcriptional DC regulation, the chemokine-mediated crosstalk between cross-presenting DCs and the cytotoxic T cells to be cross-primed and immune-escape mechanisms of pathogens.
